# Donald West, MD, LittD, FRCPsych

**DOI:** 10.1192/bjb.2020.125

**Published:** 2021-04

**Authors:** Jeffrey Weeks, Philip Graham

Formerly Director of the Institute of Criminology, Cambridge, UK

**Figure:**
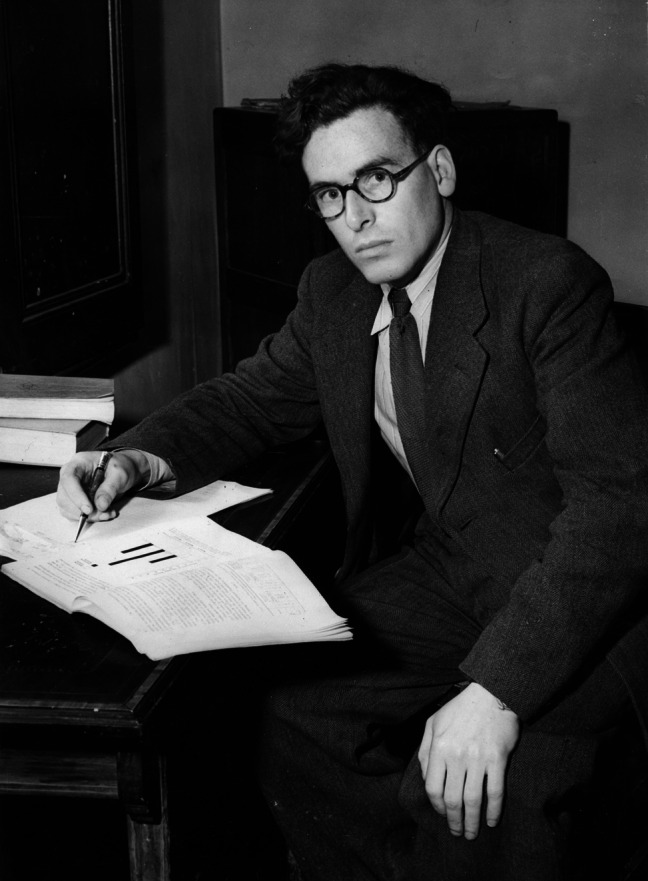
Credit: Institute of Criminology, Cambridge.

Donald West, who died on 31 January 2020 aged 95 years, made a substantial contribution to knowledge in two areas of relevance to psychiatrists. In 1955 he published *Homosexuality*, a book for the general public. This was a sober, cautious, thorough presentation of the evidence about homosexuality in history, society and psychology, with some case studies from ‘self-confessed’ homosexuals to leaven the dough. It concluded that no doctor should advise a young person to be content with his sexual orientation without a ‘grave warning’ – ‘about the frustration and tragedy that so often attend this way of life’. West's intention, as he described later in his memoir *Gay Life, Straight Work* (2012), was in fact more ambitious: to help the cause of greater understanding and legal reform, and he risked his career to do so. As he put it in his memoir, ‘For a young, unmarried professional to have stuck his neck out so recklessly seems, in retrospect, quite crazy […]. I was protected by the hypocritical medical label’. Two years later, the Wolfenden Committee, which was sitting at the time *Homosexuality* was published, set up to reconsider the law, made recommendations for modest reform.

West's book played an important part in changing the climate of opinion towards homosexuality in Britain. Many gay people of his generation and younger found it comforting to read something that confirmed their ‘normality’. It was, however, a further 10 years before male homosexuality was decriminalised in the Sexual Offences Act 1967. Subsequently, West continued to teach and conduct research on sexuality, publishing *Sexual Crimes and Confrontations: A Study of Victims and Offenders* in 1987 and (with Buz de Villiers) *Male Prostitution* in 1992. He also carried out, with Richard Green, a comparative study of controls on homosexuality across countries, published as *Sociolegal Control of Homosexuality: A Multi-Nation Comparison* (1997).

West's other contribution to knowledge was his study of juvenile delinquency. In 1961, he set up the Cambridge Study in Delinquent Development. This study became one of the major, continuing, prospective longitudinal studies internationally in the field of developmental criminology. It began as a prospective survey of 411 south London boys, aged 8 years in 1961, who have since been interviewed at intervals through their lives. Their own children have been interviewed in more recent years, enabling a rich and extensive range of findings about antecedents and causes of criminality and desistance. Working together with his colleague David Farrington, who joined him in 1969, a number of major publications have emerged, including *Who Becomes Delinquent* in 1973, *The Delinquent Way of Life* in 1977 and *Delinquency: Its Roots, Careers and Prospects* in 1982.

Donald West trained in medicine at the University of Liverpool Medical School, qualifying as a doctor in 1947. While he was studying medicine, he had developed an interest in psychical research, finding in it an alternative to the religious enthusiasms of his parents. He had a lifelong interest in the paranormal, his first post being as research officer to the Society for Psychical Research (SPR). His first book was *Psychical Research Today,* published in 1954. His sceptical attitude to findings in this field did not, however, find favour with his employers and he was advised to find employment elsewhere. Although he doubted the claims of many spiritualist enthusiasts, he was convinced that extrasensory perception had a genuine psychological basis, deserving scientific laboratory research and statistical analysis. Indeed, he remained committed to studying the paranormal throughout his career, and served several times as the president of the SPR.

He then trained in psychiatry at the Maudsley Hospital and Institute of Psychiatry, where he worked with Peter Scott, the leading forensic psychiatrist of the day. After completing his training, he worked at the Marlborough Clinic in Hampstead. In 1960 he joined the newly established Institute of Criminology in Cambridge as Assistant Director of Research. The founder and director of this institute was Sir Leon Radzinowicz, who was a strong believer in interdisciplinary research. West spent the rest of his career there, as lecturer, reader then Professor of Clinical Criminology. He was Director of the Institute from 1981 until his formal retirement in 1984. At Cambridge he became a Fellow of Darwin College, and was promoted to a personal Chair in Clinical Criminology. He also worked in an out-patient clinic at Addenbrooke's Hospital, Cambridge, as an honorary consultant psychiatrist. He was appointed to the Parole Board on its foundation in 1968 and, after his retirement from the Institute, served as a Mental Health Commissioner (1992–1997).

Donald was born in Liverpool, in a traditional red-brick workers’ house near the docks, the only child of John, a catering manager with Cunard, and Jessie. His parents were of working-class origins with high aspirations. He was a sickly child, cosseted by his religious mother, who died when Donald was 11. His father later remarried. He felt the manner of his upbringing left him with chronic shyness and feelings of inadequacy. He won a scholarship to the fee-paying Merchant Taylors’ school in Liverpool, from where went on to study medicine at Liverpool University.

His memoir was written with the encouragement of a gay writers’ workshop in a manner quite different from his academic style. For many years he attended social meetings of Opening Doors London, the leading organisation for older LGBT+ people. West appeared an austere and, in his own words, a not particularly clubbable person, but he had a dry wit and a wide and eclectic network of friends.

He was in a relationship with the art historian Pietro Raffo for more than 45 years, until Raffo's death in 2000. In 2006, he entered into a civil partnership with Vincenzo. He died on 31 January 2020. Vincenzo survives him.

This obituary is based on one published in *The Guardian* on 23 April 2020 (https://www.theguardian.com/books/2020/mar/11/donald-west-obituary).

